# Aligning Superconducting Transition-Edge Sensors by Reflected Wave Intensity Measurement

**DOI:** 10.3390/s23073495

**Published:** 2023-03-27

**Authors:** Pei-Sa Ma, Hong-Fan Zhang, Xingxiang Zhou

**Affiliations:** Department of Optics and Optical Engineering, CAS Key Laboratory of Quantum Information, and Synergetic Innovation Center of Quantum Information and Quantum Physics, University of Science and Technology of China, Hefei 230026, China

**Keywords:** sensor alignment, detection efficiency, transition-edge sensor

## Abstract

It is critical to accurately align a quantum photon detector such as a superconducting transition-edge sensor (TES) to an optical fiber in order to optimize its detection efficiency. Conventionally, such alignment requires advanced infrared imaging equipment or sophisticated microfabrication. We introduce a novel technique based on the simple idea of reflected wave intensity measurement which allows to determine the boundary of the sensor and align it accurately with the fiber. By routing a light wave through an optical fiber for normal incidence on the surface of the sensor chip, and separating the reflected wave coupled back into the fiber from the input signal with a circulator, we can observe the variation in the reflected wave intensity when the beam spot of the fiber crosses the boundary between the sensor and substrate that have different reflectivity, and adjust the position of the fiber such that its output falls on the sensor. We evaluate quantitatively the precision of our alignment method, as well as the conditions that must be met to avoid photon loss caused by light beam divergence. After demonstrating the working principle of our scheme and verifying the alignment result experimentally, we employ it for efficient input signal coupling to a TES device, which is used for photon-number-resolving measurement to showcase the successful application of our alignment method in practice. Relying on only ordinary and widely used optical elements that are easy to operate and low in cost, our solution is much less demanding than conventional methods. Dramatically easier to implement and not restricted by the detection mechanism of the sensor, it is accessible to a much broader community.

## 1. Introduction

In multi-photon optical quantum information processing [1], it is necessary to count single photons in order to measure the multi-photon quantum state of light, a very difficult task due to the extremely small energy of photons in the popular wavelength range for optical experiments. Fulfilling this challenge is superconducting transition-edge sensors (TESes) [2] which are known for their extraordinary ability to resolve photon numbers in the visible to near-infrared wavelength range [3,4,5,6,7,8,9]. Due to such unique capability in comparison with other modern quantum photon detectors, TESes are a powerful tool indispensable for multi-photon quantum information research and quantum optics experiments [10,11,12]. Since quantum photon detectors like TESes detect light field so weak that it contains only a few photons, it is critical for them to have an optimized detection efficiency (DE) as any photon loss has a significant impact on the measurement result. As signal photons are routed by an optical fiber to the TES mounted in a refrigerator with milli-Kelvin temperatures, a key requirement to improve its DE is that the sensor must be accurately aligned with the optical fiber that transmits the optical signal to be detected. Any misalignment will make the sensor fail to receive all light from the fiber and results in a reduced DE, as photons not impinging on the sensor cannot be detected. When the detector is very small, e.g., only a few microns in size, its alignment to a single-mode fiber with a core smaller than 10 μm is a nontrivial challenge, especially when bulky automatic equipment cannot be employed and in situ adjustment is not possible due to the nature and constraints of the application. Unfortunately, this difficulty is particularly pronounced for photon-number-resolving TESes, as their sizes have been reduced to the extent of being comparable to that of a single-mode fiber core in order to reach an energy resolution high enough to resolve photon numbers. The requirement that they must be operated at extremely low temperatures close to absolute zero and in an environment with as little noise as possible also excludes the use of most existing automatic aligners. Effective techniques to overcome the challenge of alignment are then critical for photon-number-resolving TESes, as well as other quantum photon detectors with an analogous requirement on alignment and similar difficulty in achieving it. In fact, their impact reaches much further, as efficient coupling to the optical signal and an optimized DE are desirable characteristics for any photon detectors.

Because of the significance of alignment, it has been an important topic in the research and development of TESes and other single-photon detectors. An early attempt to align a TES to an optical fiber [13] has relied on simple guiding structures like a silicon V-groove to position the fiber and visual inspection under a microscope to align the device. Such a procedure has limited precision due to parallax errors in observation under the microscope from the front side of the sensor chip at an oblique angle. Furthermore, to prevent the fiber end from blocking the sensor from the microscope view, it can not be positioned too close to the senor, which results in a gap over 100 μm [13] between the fiber end and the sensor. Consequently, the light beam from the fiber diverges considerably over such a large gap before it impinges on the sensor chip. This divergence can result in a beam spot appreciably larger than the sensor, which amounts to non-negligible photon loss and thus poor coupling and a reduced DE for the sensor. A more reliable approach is by through-chip imaging of the sensor and the beam spot from the back side of the chip [14,15,16], in which one can tell if they are aligned by comparing the location of their images. However, this method is quite demanding since special imaging equipment such as an inverted infrared microscope is required, due to the fact that the sensor chip is opaque to visible light. In [17], the authors offered another technique that they termed “self-aligning”, in which a circular area about the size of the inner diameter of a commercially available fiber ferrule around the sensor is etched off the wafer and fit into the fiber ferrule. When a fiber is inserted in the ferrule, it is then in alignment with the sensor located at its center. Though the authors demonstrated successful alignment of 25 μm TES devices, their technique relies heavily on advanced microfabrication as it employs a time-consuming and costly process to etch through an entire wafer to prepare a device in the right shape and size to fit in a commercial fiber ferrule. Such advanced processing, as well as necessary work for making high-precision supporting structures to position the device in the ferrule, offsets the benefit and appeal of exploiting a commercial optical fiber ferrule assembly and presents a nontrivial technical hurdle.

In order to overcome the drawbacks of conventional methods and alleviate the challenge of accurate microsensor alignment, we recently proposed and demonstrated experimentally a solution [18] based on the simple idea of making alignment structures on the surface of the sensor chip in which the fiber can be inserted to be aligned with the sensor. Our method achieves high precision without requiring any special infrared imaging equipment. It does not involve any advanced microfabrication process other than simple lithography and patterning of photoresist. Consequently, it is much less demanding on equipment and fabrication, and it has a much lower implementation cost. Still, we are interested in further exploring new techniques to push the barrier to accurate microsensor alignment even lower. In particular, we prefer an all-optical solution that employs only ordinary optical elements and well-known optical techniques and does not involve any microfabrication at all. Such a solution not only simplifies TES alignment dramatically but will be accessible to a much larger community of optical sensor users, as it allows microsensor users to solve the alignment problem using components they already own and with knowledge limited to the optics domain.

## 2. Sensor Alignment Based on Reflected Wave Intensity Measurement

### 2.1. Working Principle

**Figure 1:**
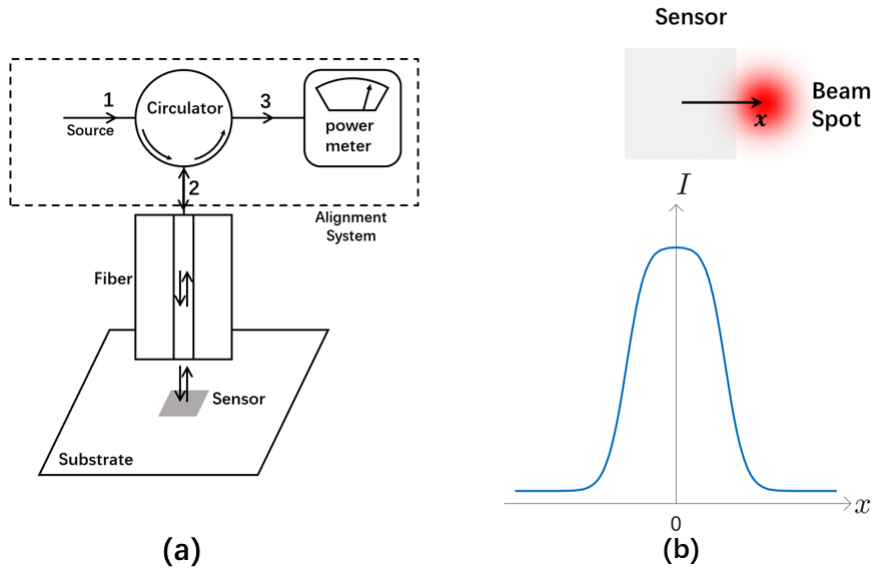
Alignment system and its working principle. (**a**) Alignment system based on the measurement of reflected wave intensity, with a single-mode fiber at the frequency of the signal wave used for alignment, a circulator in which light can only travel from port 1 to port 2, and from port 2 to port 3, and a power meter to measure the intensity of the reflected wave. (**b**) The jump in the intensity of the reflected wave when the beam spot of the fiber moves across the boundary between the sensor and the substrate, assuming the movement is along the midline between two opposite sides of a rectangular sensor.

Our alignment method is based on the simple idea that the reflected wave intensity will be different when the beam spot of the optical fiber falls on the sensor or substrate, provided that the reflectivity of the sensor material is different than that of the substrate and other structures surrounding the sensor. There are stringent requirements that must be met in order to exploit this idea for sensor alignment, though. Not only do we have to use a signal wave with appreciably different reflectivity on the sensor material and substrate, but an exquisite optical circuit must be adopted that allows us to separate the reflected wave from the input signal and guide it to a power meter for accurate intensity measurement. To help interpret and appreciate the nuances of our alignment solution, we consider the specific design shown in Figure 1a. In this setup, an input signal from the source port of the circulator is incident on the surface of the sensor chip when it exits the guiding single-mode optical fiber. After normal incidence and reflection, it returns to the fiber and travels in the opposite direction in it. In order to measure the intensity of the reflected wave, it must be separated from the input signal. This is accomplished by using an optical circulator [19] in Figure 1a, a kind of optical isolator element in which light can only travel in one direction. Because of the circulator, the reflected wave is directed to a different port than the source port in Figure 1, where we can measure its intensity by using a power meter.

If the reflectivity of the input signal on the sensor material is different than that of the substrate and other structures surrounding the sensor, we will be able to tell if the fiber output is reflected by the sensor and therefore aligned with it by the intensity of the reflected wave. In fact, when we adjust the position of the optical fiber relative to the sensor, we expect to observe a jump in the intensity of the reflected wave when the beam spot of the fiber moves across the boundary between the sensor and its neighboring structures, as shown in Figure 1b. This change can be positive or negative depending on the relative reflectivity of the sensor material and its neighboring structures, and it should be more appreciable when the difference in reflectivity is large. Obviously, this jump in the reflected wave intensity across the boundary of the sensor can be used to determine the position of the sensor and help align it with the fiber. In such a technique, the position of the beam spot relative to the sensor can be inferred by monitoring the intensity of the reflected wave. It is then possible to adjust their positions until they are aligned.

The alignment system in Figure 1a consists of ordinary and low-cost optical elements that are highly robust and reliable. The entire alignment procedure involves only observation and manipulation from the front side of the sensor chip, using well-established and widely-used optical techniques such as opto-mechanical positioning and optical signal isolation. Requiring neither advanced microfabrication [17,18] nor the use of specialized infrared imaging equipment for through-chip observation from the backside of the sensor chip [14,15,16], our alignment system in Figure 1a constitutes a technically much easier solution enabled by a simple innovative concept. It lowers the barrier to microsensor alignment substantially, and it has a much lower implementation cost than conventional methods. Therefore, it is accessible to a much larger audience.

Though the working principle for our alignment solution shown in Figure 1 is not complicated conceptually, a number of design choices must be made carefully and a few subtle technical hurdles must be addressed thoroughly in order to establish it for practical use. In the rest of this section, we focus on the following issues that are critical to our alignment scheme and prove that high precision can be achieved with appropriate choice on signal wavelength and optical mode, as well as the correct optical circuit design and system configuration.

- A key element in our alignment scheme is that the reflected wave must couple back into the guiding fiber and travel in the opposite direction in it. As depicted in Figure 1a, normal incidence and reflection on the surface of the sensor chip is the most favorable configuration for the reflected wave to couple back into the fiber efficiently. For such a condition to be met, it may be necessary to adjust the angle of incidence of the light on the surface of the sensor chip, which can be accomplished by using a tilt platform to change the orientation of the fiber relative to the sensor chip. However, a factor equally important for consideration is the inevitable divergence of the light beam when it leaves the fiber and propagates in the free space between the fiber end and the chip surface. Such divergence has an adverse effect on the coupling of the reflected wave back into the fiber. Its impact on our alignment method must be carefully assessed.
- Another necessary condition for our alignment method to work is that there should be a well-defined boundary between the sensor and the substrate or other structures surrounding it, and the reflectivity of the signal wave across the boundary must be different. Intuitively, a larger contrast in reflectivity is more favorable. Nonetheless, it should be studied how the performance of our alignment technique is affected by the contrast in reflectivity.
- Our alignment scheme takes advantage of the jump in the intensity of the reflected wave when the beam spot of the fiber crosses the boundary between the sensor and substrate to determine the location of the sensor. This jump is not infinitely sharp, however. There is a region in which the reflected wave intensity gradually changes from the value on the substrate to that on the sensor. The width of this transition is an important parameter that determines the precision of our alignment scheme. It is directly related to the intensity distribution of the fiber output. An inappropriate optical mode in the fiber like a high-order helical mode with a fragmented intensity pattern can blur this transition in reflected wave intensity and undermine the foundation of our alignment scheme. We must investigate quantitatively how the reflected wave intensity changes with the misalignment and make a comparison with experimental data to evaluate the performance of our technique.

### 2.2. Fiber Mode Profile and Output Beam Divergence

In our alignment system in Figure 1a, we use an optical fiber to guide the alignment signal wave that is single-mode at its frequency. An optical sensor can be designed and optimized to detect photons at a particular frequency or frequency range. The wavelength for the signal used for alignment does not have to coincide with the sensor’s target detection frequency. It is advantageous to use a single-mode setup for alignment, however. Under such a configuration, the distribution of the optical field at the fiber end is known and we can accurately predict key metrics in our alignment scheme such as how severe the divergence of the light beam is and how the intensity of the reflected wave changes with misalignment. Multiple modes in the fiber can lead to uncertain and fragmented intensity distribution which can increase alignment error.

**Figure 2:**
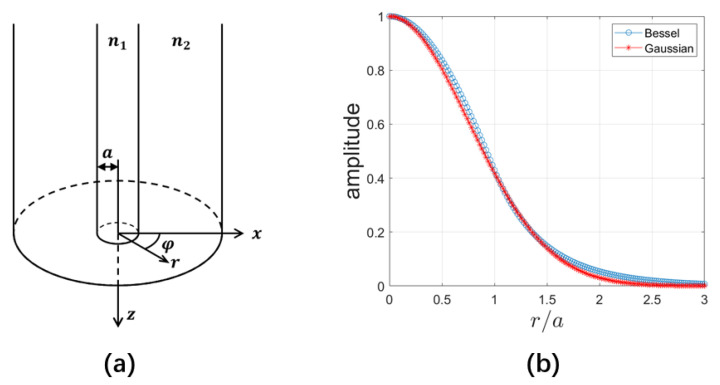
Structure of the optical fiber and its light mode. (**a**) A step-index fiber with $n_{1}$ and $n_{2}$ the index of refraction for the core and cladding. (**b**) The field amplitude of the Bessel mode in a single-mode fiber with a V number of 2.4, and its comparison with a Gaussian mode that has the same diameter where the field intensity drops to $1/e^{2}$ of the peak value. Both amplitudes are normalized to the maximum amplitude of the mode.

Solutions of optical modes in a step-index fiber shown in Figure 2a can be found in many books and references [20,21]. For the reader’s convenience, we include a brief treatment in this section. We start with the Helmholtz Equation [20]

(1) $$\nabla^{2}F+n^{2}k_{0}^{2}F=0,$$

where *F* is the electric or magnetic field, $k_{0}=2\pi /\lambda$ is the wave number in the vacuum with λ the wavelength, and $n=n_{1},n_{2}$ is the index of refraction for the fiber core and cladding. A guided mode that propagates along the fiber axis takes the form

(2) $$F\left(r,\varphi ,z\right)=f\left(r\right)e^{-il\varphi }e^{-i\beta z}$$

in the cylindrical coordinates, where *l* is the phase winding number that takes an integer value 0,±1,±2,... and β is the wave number along the fiber axis. Substituting Equation (2) into the Helmholtz Equation (1), we have

(3) $$r^{2}\frac{d^{2}f}{dr^{2}}+r\frac{df}{dr}+\left[\left(n^{2}k_{0}^{2}-\beta^{2}\right)r^{2}-l^{2}\right]f=0.$$

The solutions to Equation (3) are the Bessel functions. Considering the restriction that the field in both the core (r<a with *a* the radius of the core) and cladding should be bounded with finite energy, the solutions in the core and cladding are [20]

(4) $$f\left(r\right)\propto \left\{\begin{array}{c} J_{l}\left(\zeta r\right),\;r<a,\\ K_{l}\left(\eta r\right),\;r>a, \end{array}\right.$$

where $J_{l}$ is the *l*-th order Bessel function of the first kind, and $K_{l}$ is the *l*-th order modified Bessel function of the second kind, and [20]

(5) $$\zeta =\sqrt{n_{1}^{2}k_{0}^{2}-\beta^{2}},\;\eta =\sqrt{\beta^{2}-n_{2}^{2}k_{0}^{2}}.$$

Possible values for β and associated optical modes in the fiber can be obtained by the boundary conditions that require the electric and magnetic field to be continuous at the interface between the fiber core and cladding. For the optical fiber that we use, the core and cladding refraction index $n_{1}$ and $n_{2}$ are very close and they satisfy the weakly guiding condition $\Delta n=\left|n_{1}-n_{2}\right|\ll 1$. Under such a condition, we have the following characteristics equation [20],

(6) $$\frac{\zeta J_{l+1}\left(\zeta a\right)}{J_{l}\left(\zeta a\right)}=\frac{\eta K_{l+1}\left(\eta a\right)}{K_{l}\left(\eta a\right)}.$$

The number of solutions to Equation (6) depends on the so-called “V number” defined as follows,

(7) $$V=\sqrt{{\left(\zeta a\right)}^{2}+{\left(\eta a\right)}^{2}}=\sqrt{n_{1}^{2}-n_{2}^{2}}k_{0}=NA\cdot \frac{2\pi a}{\lambda },$$

where $NA=\sqrt{n_{1}^{2}-n_{2}^{2}}$ is the numerical aperture of the fiber. The fiber used in our alignment system has a V number smaller than the first zero of $J_{0}$, 2.405, at the frequency of the signal wave for alignment. Consequently, there is only a single mode in the fiber whose field distribution is given by

(8) $$F=\left\{\begin{array}{c} C\left[J_{0}\left(\zeta r\right)/J_{0}\left(\zeta a\right)\right]e^{-i\beta z},\;r<a,\\ C\left[K_{0}\left(\eta r\right)/K_{0}\left(\eta a\right)\right]e^{-i\beta z},\;r>a, \end{array}\right.$$

where *C* is a normalization coefficient.

The radial dependence of the single mode in Equation (8) normalized to its maximum value is shown in Figure 2b for a V number of 2.4. Shown also is the field profile of a Gaussian beam

(9) $$F_{G}=\sqrt{\frac{2}{\pi }}\frac{1}{w_{0}}e^{-r^{2}/w_{0}^{2}}e^{-i\beta z}$$

normalized to its maximum value, where $w_{0}$ has been so chosen that the intensity of the Bessel beam in Equation (8) at $r=w_{0}$ is $1/e^{2}$ of its maximum intensity. I.e., ${\left|F(r=w_{0})\right|}^{2}={\left|F(r=0)\right|}^{2}/e^{2}$. In such a specification, the value $2w_{0}$ can be considered the mode-field diameter (MFD) of the fiber. In Figure 2b, it can be seen that the Gaussian beam is a very good approximation for the single-mode Bessel beam in the fiber. It can thus be used to understand the characteristics of the fiber output and calculate the intensity of the reflected wave.

When the light exits the fiber and propagates toward the sensor, it is no longer confined by the fiber and starts to spread. If the divergence of the signal wave is large, its incidence and reflection on the sensor and substrate may not be normal, which is unfavorable for our alignment scheme. Because the single Bessel mode in the fiber is very close to a Gaussian mode, we can use the well-known Gaussian mode divergence to get a very good estimate on the spreading of the signal wave during incidence and reflection [21]. Specifically, the beam radius of a Gaussian beam at position *z* along the propagation direction is

(10) $$w\left(z\right)=w_{0}\sqrt{1+{\left(\frac{z}{z_{R}}\right)}^{2}},$$

where $w_{0}$ is the narrowest beam radius at z=0 where the beam waist is located, and

(11) $$Z_{R}=\frac{\pi w_{0}^{2}}{\lambda }$$

is the Rayleigh range over which the cross-sectional area within the beam radius doubles.

**Figure 3:**
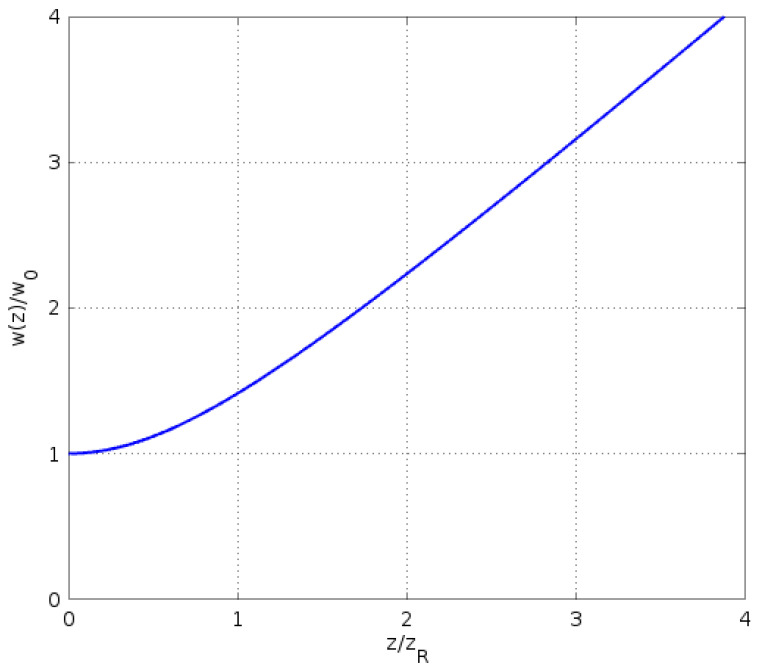
The beam radius of a Gaussian beam along the direction of propagation.

In Figure 3, the beam radius in Equation (10) is plotted. It can be seen that it increases linearly with *z* at a large distance $z>>z_{R}$, indicating that the beam diverges at a half angle of $\lambda /(\pi w_{0})$ in the far field. In the near field $z\ll Z_{R}$, however, the beam radius changes little from the value at the beam waist, $w_{0}$. Consequently, if the distance *d* between the fiber end and the sensor in Figure 1a satisfies the condition

(12) $$2d\ll Z_{R},$$

then the incidence and reflection of the signal wave on the sensor chip is normal. For the signal wavelength and fiber MFD used in our alignment system, $Z_{R}\approx 50\;\mu$m. In the alignment, the fiber end is positioned only a few microns from the surface of the sensor chip, and the condition in Equation (12) is very well satisfied. Therefore, the incidence and reflection of the signal wave can be considered normal to the chip surface, which is favorable for our alignment scheme.

### 2.3. Reflected Wave Intensity

**Figure 4:**
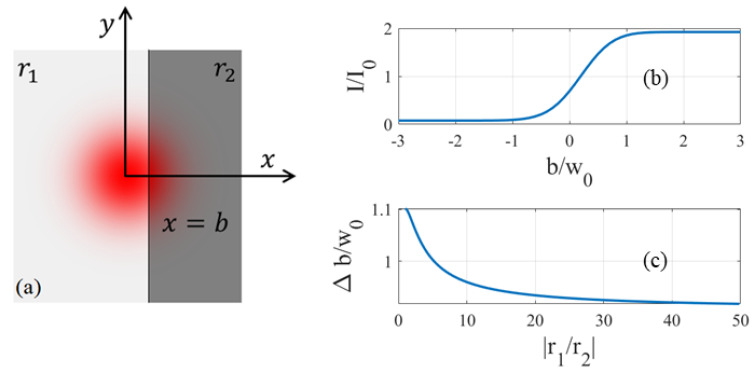
Change in reflected wave intensity across the boundary between two regions of different reflectivity. (**a**) The beam spot of the fiber output that falls on the boundary between two regions of different reflectivity on the surface of the sensor chip. (**b**) Intensity of the reflected wave as a function of *b*, the distance of the beam center from the boundary line, measured in units of the average intensity in the two regions, $I_{0}$. The amplitude reflection coefficient $r_{1}=5r_{2}$, and $w_{0}$ is the field mode radius of the fiber. (**c**) The transition width in the reflected wave intensity as a function of $\left|r_{1}/r_{2}\right|$.

As proved in Section 2.2, when we position the fiber end from the surface of the sensor chip at a distance much smaller than the Rayleigh range, the incidence and reflection of the signal wave are normal to the chip surface. The propagation of the light beam between the fiber end and the sensor, as well as its coupling back into the fiber, can be treated without divergence. At such a small distance, the sensor is blocked from the microscope view by the fiber when we observe from the front side of the chip. We must rely on the intensity of the reflected wave to determine if the sensor is aligned with the fiber output. To explain quantitatively how this is possible, we use the simple model in Figure 4a to study the change in the intensity of the reflected wave when the beam spot of the fiber moves across the boundary between two regions of different reflectivity on the sensor chip. In Figure 4a, the line at x=b separates the two regions that extend infinitely in the *y* direction. When we move the fiber parallel to the xy plane in an alignment procedure, its beam spot may fall in either region, or partially in both regions, depending on the value of *b* which characterizes how far the beam center is from the boundary between the two regions. Assuming a Gaussian output $Ae^{-r^{2}/w_{0}^{2}}$ from the fiber and normal incidence and reflection on the surface of the sensor chip, we can calculate the reflected field when it arrives at the fiber end as follows,

(13) $$F_{r}=Ae^{-r^{2}/w_{0}^{2}}\left(r_{1}u\left(b-x\right)+r_{2}u\left(x-b\right)\right)e^{-i2k_{0}d},$$

where $r_{1}$ and $r_{2}$ are the amplitude reflection coefficient in both regions, and

(14) $$u\left(x\right)=\left\{\begin{array}{cc} 0,\; & x<0,\\ 1,\; & x\geq 0, \end{array}\right.$$

is the step function. The reflected wave $F_{r}$ in Equation (13) is no longer a Gaussian mode as long as $r_{1}\neq r_{2}$. Consequently, it cannot fully couple back into the single-mode fiber. Only the portion in the same profile with the single mode in the fiber, approximated by the Gaussian mode in Equation (9), can enter the fiber in the opposite direction and propagate to the power meter which measures its intensity. The rest is lost due to mode mismatch. Therefore, the intensity *I* of the reflected wave that couples back into the fiber can be obtained by calculating the overlap between the reflected field $F_{r}$ and the fiber mode $F_{G}$, i.e., the probability of $F_{r}$ being in $F_{G}$. We have

(15) $$\begin{array}{cc} I= & {\left|\int_{-\infty }^{\infty }\int_{-\infty }^{\infty }F_{r}\cdot F_{G}^{*}dxdy\right|}^{2}\\ = & \frac{2}{\pi }\frac{A^{2}}{w_{0}^{2}}{\left|r_{1}\int_{-\infty }^{b}dx\int_{-\infty }^{\infty }dy\cdot e^{-2r^{2}/w_{0}^{2}}+r_{2}\int_{b}^{\infty }dx\int_{-\infty }^{\infty }dy\cdot e^{-2r^{2}/w_{0}^{2}}\right|}^{2}\\ = & A^{2}{\left|\frac{r_{1}+r_{2}}{2}+\frac{r_{1}-r_{2}}{2}\mathrm{erf}\left(\frac{\sqrt{2}b}{w_{0}}\right)\right|}^{2}, \end{array}$$

where

(16) $$\mathrm{erf}\left(x\right)=\frac{2}{\sqrt{\pi }}\int_{0}^{x}e^{-t^{2}}dt$$

is the error function. In deriving the reflected wave intensity in Equation (15), we have neglected the reflection at the fiber end when the light exits and couples back into the fiber. This reflectivity is only a couple percent and does not have a material impact on the conclusions of our analysis. For materials involved in TES devices, the phase of the reflection coefficient is close to π. For simplicity, we then assume $r_{1}$ and $r_{2}$ are in phase in subsequent studies.

In Figure 4b, the intensity of the reflected wave is plotted as a function of *b* for a 5-time difference in the amplitude reflection coefficient between the two regions. We see that the intensity of the reflected wave is a constant when |b| is large. In this case, the beam spot is located entirely in one of the two regions and the intensity of the reflected wave is determined by its reflectivity, ${\left|r_{1}\right|}^{2}$ or ${\left|r_{2}\right|}^{2}$. We can tell which region the beam spot is in by measuring the reflected wave intensity. A larger difference in the reflectivity increases the contrast $\left|{\left|r_{1}\right|}^{2}-{\left|r_{2}\right|}^{2}\right|/\left({\left|r_{1}\right|}^{2}+{\left|r_{2}\right|}^{2})\right)$ between the reflected wave intensity in the two regions. When |b| is comparable to $w_{0}$, in contrast, the boundary between the two regions is so close to the beam center that the light shines on both regions. In this case, the light beam is reflected by both regions and the intensity of the reflected wave changes from the value in one region to that in the other over a range comparable to $w_{0}$. By sweeping the beam spot across the two regions and observing the change in the reflected wave intensity, we can determine the location of the boundary line with uncertainty up to the transition width of the reflected wave intensity in Figure 4b. Without loss of generality, we assume $\left|r_{1}/r_{2}\right|>1$, and denote the intensity of the reflected wave in the two regions $I_{1}\propto {\left|r_{1}\right|}^{2}$ and $I_{2}\propto {\left|r_{2}\right|}^{2}$. If we define the transition width Δb in Figure 4b by the difference between $b_{1}$, the value at which *I* is within $\left(I_{1}-I_{2}\right)/e^{2}$ of $I_{1}$, and $b_{2}$, the value at which *I* is within $\left(I_{1}-I_{2}\right)/e^{2}$ of $I_{2}$, we have $\Delta b=\left|b_{1}-b_{2}\right|$ with

(17) $$\begin{array}{c} {\left|\frac{r_{1}+r_{2}}{2}+\frac{r_{1}-r_{2}}{2}\mathrm{erf}\left(\frac{\sqrt{2}b_{1}}{w_{0}}\right)\right|}^{2}={\left|r_{1}\right|}^{2}-\left({\left|r_{1}\right|}^{2}-{\left|r_{2}\right|}^{2}\right)/e^{2}, \end{array}$$

(18) $$\begin{array}{c} {\left|\frac{r_{1}+r_{2}}{2}+\frac{r_{1}-r_{2}}{2}\mathrm{erf}\left(\frac{\sqrt{2}b_{2}}{w_{0}}\right)\right|}^{2}={\left|r_{2}\right|}^{2}+\left({\left|r_{1}\right|}^{2}-{\left|r_{2}\right|}^{2}\right)/e^{2}. \end{array}$$

The values for $b_{1}$, $b_{2}$, and Δb can be obtained by solving these equations numerically. In Figure 4c, we plot the calculated values of Δb as a function of the ratio between $r_{1}$ and $r_{2}$. It is seen that the transition width is comparable to $w_{0}$, which is a manifest of the fact that the variation in the reflected wave intensity is dictated by the intensity profile of the optical mode in the fiber. It has a weak dependence on the ratio $\left|r_{1}/r_{2}\right|$. In the high-contrast limit characterized by a large disparity between the reflectivity in both regions, it approaches a constant value of

(19) $$\Delta b\approx 0.91w_{0}.$$

For the fiber used in our alignment which has an MFD of about 9.2 μm at a wavelength of 1310 nm, Δb≈4.2μm.

**Figure 5:**
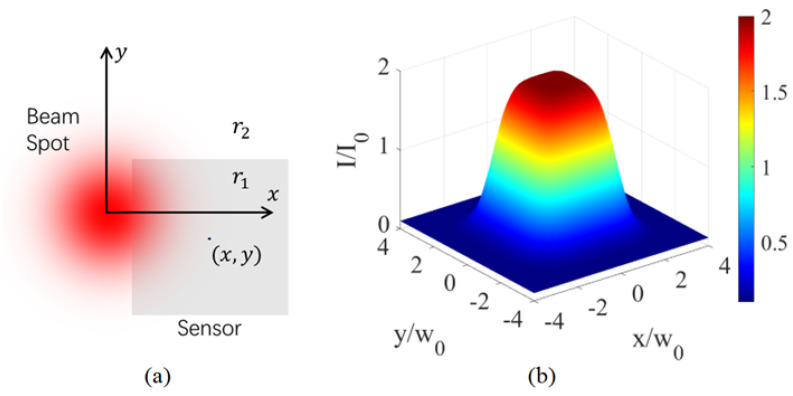
Intensity of reflected wave versus misalignment between a square sensor and the beam spot. (**a**) The beam spot of the fiber output and a sensor that has a different reflectivity than its surrounding substrate. (**b**) Intensity of the reflected wave as a function of the misalignment between the beam center and the center of the sensor, measured in units of the average intensity when the beam is entirely reflected by the sensor and the substrate. The reflectivity $r_{1}=4.5r_{2}$, the size of the square sensor is $4w_{0}\times 4w_{0}$, $w_{0}$ being the mode field radius of the fiber.

The analysis based on the model in Figure 4a can be generalized to an enclosed area such as a sensor which has a different reflectivity than the substrate and other surrounding structures, as shown in Figure 5a. By the same argument that leads to Equation (15), we can write the intensity of the reflected wave as follows,

(20) $$I=\frac{2}{\pi }\frac{A^{2}}{w_{0}^{2}}{\left|r_{1}\int \int_{sensor}dxdy\cdot e^{-2r^{2}/w_{0}^{2}}+r_{2}\int \int_{substrate}dxdy\cdot e^{-2r^{2}/w_{0}^{2}}\right|}^{2},$$

where $r_{1}$ and $r_{2}$ are the amplitude reflection coefficient of the sensor and substrate and the two integrals are performed over the sensor and substrate area, respectively. Obviously, the result depends on both the separation between the sensor and the beam spot and their relative orientation. Unlike in Figure 4a, the shape of the overlap between the sensor area and the beam spot can be irregular and the intensity of the reflected wave cannot be evaluated analytically. Nonetheless, we can calculate it numerically as a function of the mismatch between the beam center and the center of the sensor.

In Figure 5b, the reflected wave intensity of a square sensor obtained by numerical calculation based on Equation (20) is plotted as a function of its misalignment with the beam center. We see that the intensity of the reflected wave jumps along the perimeter of the sensor due to the different reflectivity of the sensor and substrate. The transition width is comparable to $w_{0}$ as in Equation (19). By moving the fiber in the vicinity of the sensor and observing the change in the reflected wave intensity, we can infer how well the sensor is aligned with the fiber output. As long as the size of the sensor is comparable to or larger than $2w_{0}$, the MFD of the single-mode fiber, there is a range for the fiber position within which the reflected wave intensity approaches the value when the beam spot is reflected entirely by a film in the sensor material. Within this range, the reflected wave intensity stays nearly constant because almost all light is reflected by the sensor. The sensor can then be aligned with the fiber by adjusting the fiber position to within this range using a translation stage. Notice that, in order for the sensor to receive most light coming out of the fiber which is necessary for realizing a high DE, the sensor must be larger than the MFD of the fiber to cover the area where most of the light energy falls. It is always possible to use our method to align such a sensor.

## 3. Experimental Verification

**Figure 6:**
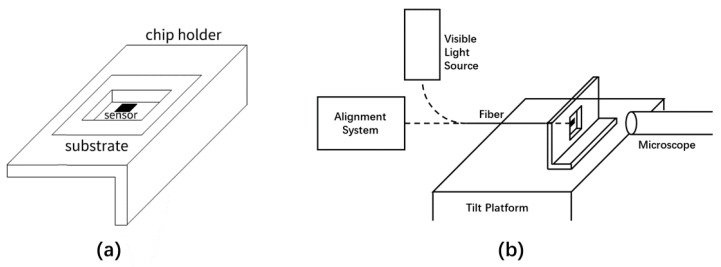
Alignment and verification with a dummy sensor on a transparent substrate [18]. (**a**) Dummy sensor fabricated on a substrate transparent to visible light and placed in a through-hole on a chip holder. (**b**) Setup to examine the position of the beam spot and compare it against that of the sensor by side-way observation from the backside of the sensor chip using an ordinary microscope under visible light. The fiber is connected to the alignment system in Figure 1a operating at 1310 nm during alignment. It is attached to an (unshown) 3-axis translation stage to adjust its position against the sensor chip. When the alignment is completed, the fiber is connected to a visible light source to verify the result.

In order to verify the working principles of our alignment scheme and evaluate its accuracy, we employ a technique shown in Figure 6 that was introduced in our earlier work [18]. In this technique, we fabricate dummy devices on a substrate transparent to visible light and glue the sensor chip on a chip holder that has a through-hole in it, at such a position that the sensor device is located in the hole, as shown in Figure 6a. We then attach the chip holder to a tilt platform with the sensor chip facing sideways. By coupling a bright visible light in the fiber, we can use an ordinary microscope placed horizontally to observe the sensor and the beam spot from the backside of the chip under visible light via the hole in the chip holder and through the transparent substrate, as shown in Figure 6b. This verification method does not require special imaging equipment such as an inverted infrared microscope for through-chip observation [14], yet it offers higher quality images which are favorable for evaluating the alignment result by checking the position of the beam spot against that of the sensor. It is therefore a very effective verification scheme that is easier to realize and much lower in cost.

In Figure 6b, we extend the verification system in [18] to also use it for experimentally checking the theoretical and numerical results in Section 2. This is accomplished by connecting the fiber to the alignment system in Figure 1a during the alignment phase, as depicted in Figure 6b. The fiber is prepared from a fiber optic pigtail with a connector on the input end which allows coupling an optical signal in the fiber easily. We also attach the fiber to a 3-axis translation stage to adjust its position during alignment. In our alignment experiment, we use a G.652D single-mode fiber at 1310 nm which has an MFD of 9.2 μm. The frequency of the laser source and circulator in the alignment system is also 1310 nm. Once the alignment is done, we disconnect the fiber from the alignment system and reconnect it to a visible light source to check the result of alignment, using the method explained earlier.

**Figure 7:**
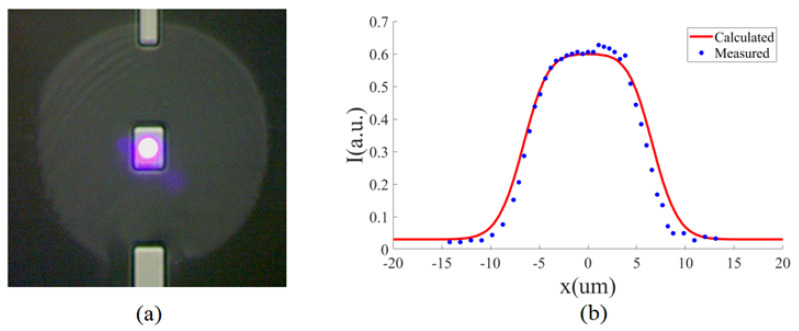
Experimental verification of alignment by reflected wave intensity measurement. (**a**) Image of a 15 μm × 20 μm dummy sensor fabricated on a transparent quartz substrate, and that of the beam spot of the fiber when it is aligned with the sensor by reflected wave intensity measurement, photographed using the verification system in Figure 6. (**b**) Measured reflected wave intensity and calculated curve based on Equation (20), against misalignment along the midline of the device, representative of the degree of agreement between experimental data and numerical results. The ratio between the amplitude reflection coefficient on the device and substrate is measured to be 4.47.

Shown in Figure 7a is a dummy sensor photographed using the verification system in Figure 6. The dummy sensors used in our experiments are fabricated on quartz substrates using the same process and lithography masks for our TES devices described in Section 4 and therefore in the same layouts, except that lead wires are omitted since they are irrelevant for verifying the results in Section 2. Dummy devices in different sizes are fabricated and tested in our experiments to evaluate the performance of our alignment method. The layout shown in Figure 7a is for a 15 μm square device, which is several microns longer in one direction than its intended size to leave room for the lead wires to make contact. Without lead wires to cover the edge of the device, the dummy sensor has a dimension of 15 μm × 20 μm. As the dummy sensors are used to verify our alignment scheme, not to detect photons, they do not have to be in the same material as our TES devices. In our experiments, they are made of titanium and aluminum thin films which have good adhesion properties on the quartz substrate.

In the alignment experiment, we first roughly position the fiber end near the sensor using the translation stage it is attached to. In this process, we move the fiber end to just a few microns from the surface of the sensor chip in order to satisfy the condition in Equation (12) to avoid divergence of the signal beam. We then tune the tilt stage in Figure 6b and adjust the angle of the fiber with respect to the sensor chip to ensure that the incidence and reflection of the signal wave are normal to the chip surface. We can tell when normal incidence and reflection are achieved by monitoring the intensity of the reflected wave when we tune the angle of the tilt stage, as the intensity should be the maximum under normal incidence and reflection. Next, we use the translation stage to move the fiber end around the sensor area in a plane parallel to the chip surface to adjust its lateral misalignment with the sensor, and measure the intensity of the reflected wave as a function of the position of the fiber during this process. The recorded data can be compared with the calculation results like those in Figure 5 to verify the working principles of our alignment method and evaluate its performance.

When we move the fiber end to adjust its lateral misalignment with the sensor in both the *x* and *y* directions, the measured reflected wave intensity has a profile like that in Figure 5b with a plateau over the sensor area in the same shape as the sensor. We compare the measured data with the calculated results based on the sensor dimensions. Since it is very difficult to visualize both the calculated and measured reflected wave intensity in the same 3d plot and compare them, for clarity we show in Figure 7b the measured intensity and numerically calculated curve against misalignment along the midline of the device between the midpoints of its lengths, which is representative of the degree of agreement between measured and calculated values. The jump in the intensity across the boundaries of the sensor is clearly seen, and good agreement between the measured data and calculated result is observed. In particular, the experiment demonstrates that the transition width in the reflected wave intensity is indeed comparable to the mode field radius $w_{0}$ of the fiber as in Equation (19), thus proving that our method can accurately align a sensor comparable to or larger than the MFD of the fiber. We repeat the experiment with dummy sensors in different sizes between 5 μm and 20 μm and obtain similar results as long as the sensor size is comparable to or larger than the MFD of the fiber. When the sensor is considerably smaller than the MFD of the fiber, we no longer have a plateau on the top of the measured intensity profile, as a sensor smaller than the cross sectional area of the optical mode in the fiber can not reflect all light in it.

To align the sensor to the fiber output, we adjust the position of the fiber in both the *x* and *y* direction until the reflected wave intensity falls within the plateau on the top of the intensity distribution. For a rectangular sensor, we perform this procedure along both its length and width such that the beam spot is located in the 2d plateau on the top of the reflected wave intensity profile as shown in Figure 5b. Once this is done, we can conclude that the sensor is aligned with the fiber output and we can connect the fiber to a visible light source to verify the alignment result, as illustrated in Figure 6b. Shown in Figure 7a is a micrograph of the beam spot, produced by coupling a bright visible laser light into the fiber, and the sensor, after it is aligned to the fiber by measuring the reflected wave intensity. Indeed, our method based on reflected wave measurement results in very high-precision alignment, without using any special imaging equipment for infrared through-chip observation [14,15,16] or relying on any micro fabrication process [17,18].

## 4. Application in TES

TES is a superconducting sensor with a very low critical temperature that detects photons based on the resistance change caused by the heat of absorbed photons when the device is biased in its superconducting to normal transition with the help of nontrivial negative electrothermal feedback [2]. Because of the small heat capacitance of the device at its very low critical temperature, it can reach a very high energy resolution that is sufficient to resolve photon numbers in the visible to near-infrared range, an extraordinary and unique capability that makes it very valuable for multi-photon quantum information tasks and quantum optics experiments. The basic structure of a TES is a thin film consisting of a superconducting metal layer, and possibly one or more normal metal layers to lower the device’s critical temperature through the proximity effect [2] when the thickness of the normal layer is increased. Since the superconducting and normal metal layers have a reflectivity appreciably higher than that of the substrate, usually in silicon nitride or silicon oxide, and the TES has well-defined boundaries from the substrate and other surrounding structures like the lead wires, our alignment method based on the reflected wave intensity measurement is well suited for TES devices.

**Figure 8:**
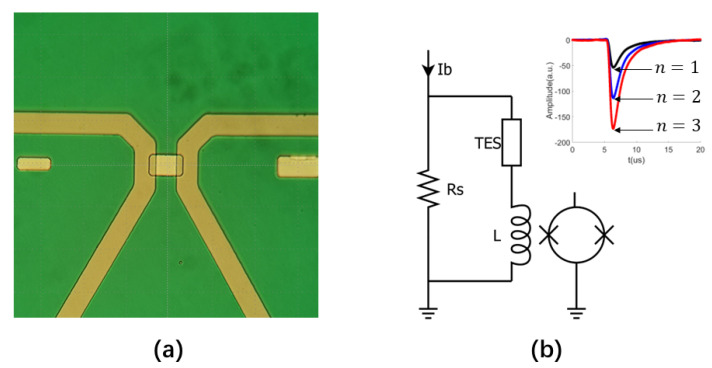
Photon counting experiments based on Titanium/Silver TES aligned by reflected wave intensity measurement. (**a**) Micrograph of a fabricated TES device. (**b**) A voltage-biased TES circuit, realized using a current source $I_{b}$ and a shunt resistance Rs that is much smaller than the TES resistance. The current change in the TES due to absorbed photons is measured by an inductively coupled SQUID. Shown in the inset are the averaged response curves for different absorbed photon numbers.

We have developed titanium/silver double-layer TES devices on SiN substrates for application in multi-photon quantum optics experiments around 830 nm. To satisfy the requirements of these experiments, the sensor should have a sufficiently high energy resolution to resolve photon numbers at the target wavelength, a high counting rate of up to 100 kHz, and a decent DE. To meet these specifications, we must carefully choose the device parameters as optimization of different performance metrics often poses conflicting requirements. For instance, lowering the critical temperature of the TES by increasing the thickness of the normal metal layer can increase the energy resolution of the device, but it also makes its response time slower and thus its counting rate lower. As far as the sensor size is concerned, a smaller device is favorable for achieving a higher energy resolution, however a device smaller than the MFD of the fiber will fail to receive all light from the fiber leading to a lower DE. Considering these constraints, we make necessary compromises and choose the relative thickness of the silver and titanium layer in our device such that the resulting TES has a critical temperature of about 200 mK, which can offer both a sufficient energy resolution and a satisfactory counting rate for the intended application. We also use 10 μm × 10 μm devices which are only slightly larger than the MFD of the single-mode fiber to both increase the energy resolution and maximize the DE. In Figure 8a, a micrograph of the fabricated device is shown, together with a voltage biased circuit to operate the TES in Figure 8b. The fabrication process of the TES and characterization of its properties will be reported elsewhere. In this work, we focus on the alignment process of the TES device using our method based on reflected wave intensity measurement, and verification of the alignment result through DE measurement in an optical experiment using a coherent laser source.

**Figure 9:**
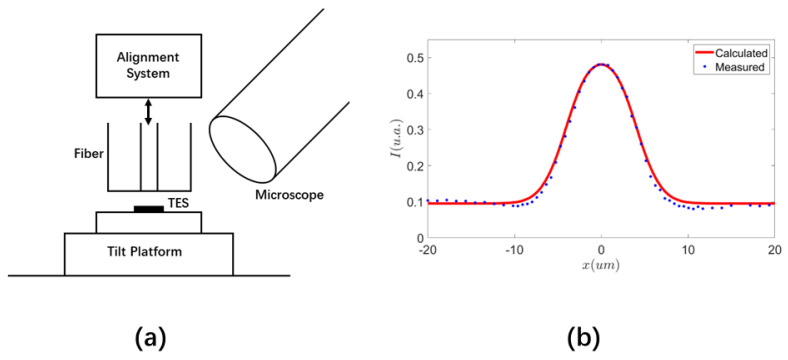
Alignment of a TES by reflected wave intensity measurement. (**a**) Alignment setup for the TES device. The fiber is connected to the alignment system in Figure 1a. It is also attached to an (unshown) 3-axis translation stage to adjust its position relative to the sensor chip. (**b**) Comparison of experimentally measured reflected wave intensity and numerically calculated curve, against misalignment along the midline of a 10 μm × 10 μm TES device, representative of the degree of agreement between experimental and numerical results. The ratio between the amplitude reflection coefficient on the device and substrate is measured to be 2.29.

Shown in Figure 9a is the setup we use to align our TES device to the output beam of a G.652D single-mode fiber at 1310 nm with an MFD of 9.2 μm. The TES device slightly larger than the fiber’s MFD is placed on a tilt platform that sits on a level stand. The fiber is connected to the alignment system in Figure 1a operating at 1310 nm. We also use an ordinary microscope to observe from the front side of the chip. With its help, we can roughly position the fiber end near the TES device by using the 3-axis translation stage it is attached to. In this process, we move the fiber end to within just a few microns from the surface of the TES chip to avoid divergence of the signal beam. In doing so, there is a risk of damaging the TES device due to accidental contact between the fiber end and the sensor. This risk can be avoided by using proximity spacers introduced in [18], which are microstructures fabricated on the surface of the sensor chip that can stop the fiber end from making contact with the sensor when it moves toward the sensor. In our devices, the proximity spacers are the lead wires which have a height greater than the thickness of the TES. When the fiber end moves toward the sensor, it cannot advance further when it makes contact with the lead wires, which protects the sensor from accidental damage caused by abrasion.

Once the fiber end is within just microns of the sensor, the sensor is blocked by the fiber from the microscope view. From this point on, the microscope is of no further use to us and we solely rely on the reflected wave intensity measurement for the remaining steps of the alignment which are essentially the same as in Section 3 when we align a dummy sensor. Specifically, we tune the angle of the tilt stage to make sure that the incidence and reflection of the signal wave on the TES chip are normal to its surface. We then move the fiber end in a plane parallel to the chip surface around the sensor area to adjust its lateral misalignment with respect to the sensor, and measure the intensity of the reflected wave against the fiber position to observe the intensity jump across the boundary of the TES. Like in the dummy sensor alignment in Section 3, we show in Figure 9b the measured intensity and numerically calculated curve against misalignment along the midline of the device, which is representative of the degree of agreement between measured and calculated values. By adjusting the fiber along both the length and width of the device to such a position that the reflected wave intensity falls in the 2d plateau on the top of the reflected wave intensity profile, we can then align the TES to the fiber output as explained in Section 3. Once the alignment is achieved, we can push the fiber end toward the sensor until it is in contact with the proximity spacers [18] to reduce the gap between them and improve the coupling further. At last, we apply a photo-sensitive epoxy and cure it with UV light to glue the fiber to the TES chip. Alternatively, we can apply the epoxy first and perform the alignment with the fiber end in the epoxy, and shine a UV light to cure the epoxy after the alignment.

**Figure 10:**
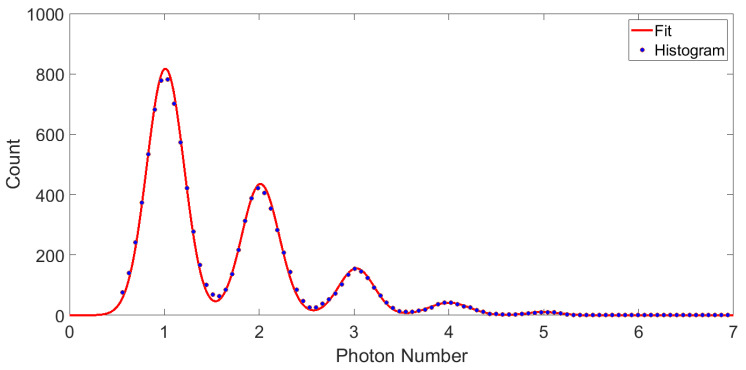
Amplitude histogram of the photon response curves and its fit by the Poissonian emission distribution function (0-photon peak not shown).

Unlike in Section 3, when the alignment is finished, we cannot verify the result by inspecting the positions of the beam spot and the sensor from the backside of the chip. This is because the SiN substrate of the TES chip is opaque to visible light, and we cannot make infrared through-chip observation without using an inverted infrared microscope. Instead, we measure the DE of the TES in an optical experiment at the device’s intended working frequency. In this experiment, heavily attenuated laser pulses with a repetition rate up to 300 kHz from an 830 nm LED are coupled into the TES through the fiber. The TES is then subject to a coherent light field which is a superposition of Fock states in different photon numbers. In each absorption event, it can measure a certain photon number with a probability determined by the weight of the corresponding Fock state in the coherent light. The photon absorption event is detected by measuring the change in the TES current caused by the absorbed photons using a SQUID inductively coupled to the TES circuit, as shown in Figure 8b. In the inset of Figure 8b, the average response curves for different photon numbers are shown. According to a double exponential fit [2], the rising and falling time constant of the response curves are 0.61μs and 1.33μs, and the device can be operated at a counting rate over 100 kHz. In Figure 10, the histogram for the amplitude of the response curves collected in an optical experiment is plotted. The height of peaks at each photon number represents the weight of the corresponding Fock state in the coherent light. The histogram in Figure 10 exhibits clear photon number resolution at the target wavelength, indicating that our device has a sufficiently high energy resolution for its intended application. Shown also in Figure 10 is the fit according to a Poissonian emission function. From the standard deviation of the fit, we obtain an FWHM energy resolution of 0.68 eV, without any filtering and smoothing of the experimental data, which is sufficient for resolving photon numbers at 830 nm or 1.49 eV. The fit also yields the mean photon number of the light field detected by the TES. Using this detected mean photon number, and the input power to the refrigerator’s fiber port measured by a carefully calibrated setup, we obtain a DE of 51%. It is consistent with the value measured by other authors with a titanium/gold TES aligned by through-chip imaging of the sensor and beam spot using an infrared microscope [16], which suggests that our alignment based on reflected wave intensity measurement is just as accurate. Therefore, we can accurately align our TES device to the fiber output using our method without special through-chip infrared imaging equipment, a significant advantage both in terms of alleviating the technical barrier and lowering the implementation cost.

## 5. Discussion

From the calculation results shown in Figure 4 and Figure 5, and the experimental data reported in Figure 7 and Figure 9, it is convincing to conclude that our alignment scheme depicted in Figure 1 is capable of accurately aligning a sensor comparable to or larger than the MFD of the fiber to achieve efficient coupling to the fiber output and a high DE for the sensor. In order to apply our alignment method, there should be a well-defined boundary between the sensor and the substrate and other surrounding structures, and a reasonable contrast in the reflectivity within and beyond the sensor area. In some sensor designs, a multi-layer optical resonance structure is grown on the sensor [15] to increase its DE by making the light pass the sensor multiple times when it bounces in the resonator. For such a sensor chip, the reflectivity is quite low at the sensor’s target working wavelength. Nonetheless, our method can still be employed to align such a sensor by using a signal wave at a different wavelength in the alignment, as long as the used signal wave has decent and different reflectivity on the sensor and substrate at its chosen wavelength. In order to increase the reflectivity contrast within and beyond the sensor, one may also purposefully fabricate surrounding structures that have appreciably different reflectivity than the sensor at the wavelength of the signal wave used for alignment.

The polarization of the signal wave used for alignment can be a factor of concern when the reflectivity on the sensor material is polarization-dependent. When the signal wave propagates in a long-haul fiber, its polarization will be randomized. This can lead to an unstable reflected wave intensity on materials with polarization-sensitive reflectivity which has an adverse impact on the alignment. We do not observe polarization-caused issues in our alignment experiments. When such a problem does arise, it is easy to deal with it by using a polarized signal wave and a short-reach single-mode fiber in the alignment. Indeed, most alignment does not need to be performed remotely. When a relatively long propagation distance is unavoidable, it can still be mitigated by using a polarization-maintaining fiber.

For sensors that do not have a well-defined boundary from their surroundings, it is possible to fabricate a marker structure such as a highly reflective metal layer beneath the sensor area, and isolation dielectric layers between the sensor and marker if necessary. We can then couple the light to the sensor by using our method to align the marker structure to the fiber output. Therefore, our alignment scheme is quite flexible and versatile, provided that necessary measures are taken to make it suit the specific application.

Obviously, though our alignment technique has certain requirements on the geometric and optical properties of the sensor, it doe not depend on its detection mechanism. Hence, it can be used for many types of microsensors though we experimentally demonstrated its application with a TES. Furthermore, though our analysis is based on an optical fiber, it does not pose a restriction on the alignment principle based on reflected wave intensity measurement which applies to other types of waveguides just as well. Therefore, both our alignment system and its underlying principle are quite general and can benefit many sensor applications.

## 6. Conclusions

We have presented a novel alignment method for microsensors that is based on the measurement of reflected wave intensity. By making the light guided by a single-mode fiber couple back into the fiber after normal incidence and reflection on the surface of the sensor chip, and monitoring the change in the reflected wave intensity when the beam spot moves across the boundary of the sensor, we can determine the location of the boundary with an uncertainty up to the mode field radius of the fiber, and align a sensor comparable to or larger than its MFD. The novelty of our scheme lies not only in its simple and innovative underlying principle that allows accomplishing the alignment without visual inspection of the sensor blocked by the fiber end under a microscope, but the practical benefits it offers in overcoming the technical drawbacks of previous methods [13,14,15,16,17,18]. Specifically, our scheme is much less demanding technically and does not require any special through-chip infrared imaging equipment [15,16] or advanced microfabrication [17,18]. Involving only ordinary optical elements that are very reliable, easy to operate, and low in cost, our solution enables high-precision alignment with a significantly lower barrier than conventional methods, and thus is accessible to a broader community. Though we demonstrated its application with a TES device, it can be adopted in many sensor technologies provided necessary measures are taken to cater to the specific requirement of the application.

## Data Availability

Data underlying the results presented in this paper are not publicly available at this time but may be obtained from the authors upon reasonable request.

## Abbreviations

The following abbreviations are used in this manuscript:

DE	Detection efficiency
eV	electron Volt
FWHM	Full width half maximum
LED	Light emitting diode
mK	milli Kelvin
SQUID	superconducting quantum interference device
TES	transition-edge sensor
UV	Ultra violet
